# Mindfulness and Empathy: Mediating Factors and Gender Differences in a Spanish Sample

**DOI:** 10.3389/fpsyg.2020.01915

**Published:** 2020-08-04

**Authors:** Raquel de la Fuente-Anuncibay, Ángela González-Barbadillo, Delfín Ortega-Sánchez, Juan Pablo Pizarro-Ruiz

**Affiliations:** ^1^Department of Science Education, Faculty of Education, University of Burgos, Burgos, Spain; ^2^Department of Specific Didactics, Faculty of Education, University of Burgos, Burgos, Spain

**Keywords:** mindfulness, empathy, mediating effect, gender, FFMQ, TEQ

## Abstract

Numerous research studies link mindfulness training to improved empathy. However, few studies focus on the mediating factors of empathy. This work has three objectives: (a) to analyze the possible mediation of mindfulness as a feature in this relation, (b) to analyze the mindfulness factors that mediate in the increase of empathy and (c) to analyze the moderating role of gender. The sample was composed of 246 Spanish-speaking university students (*M* = 24.08 years, *SD* = 8.43). The instruments used were the Five Facet Mindfulness Questionnaire (*FFMQ*) and the Toronto Empathy Questionnaire (*TEQ*). For data analysis, the indirect effect was calculated using 10000 bootstrap samples for the bias-corrected bootstrap confidence intervals (*BCI*). The improvement of empathy is mediated by the changes in mindfulness trait (B = 0.233, *p* < 0.001), disappearing in the presence of this mediator, the direct effect of mindfulness practice on empathy (B = 0.161, *p* = 0.394). We did not find a differential functioning of this mediation according to gender. *Observing* and *describing* are the *FFMQ* factors that mediate significantly between mindfulness practice and empathy.

## Introduction

Mindfulness has experienced a boom in recent years with interventions that have proven effective in different contexts and ages (Coholic and Eys, 2015; Ted Ng et al., 2016; Zeng et al., 2017). Health-related areas are those, either as a complementary therapy to treatment or as an improvement in general well-being, on which the main research in this field has been focused, for example, the importance of a normalizing, accepting, non-judgmental attitude to decrease anxiety and depression, and to foster wellbeing, reduce stigma and prejudice (Barcaccia et al., 2019; Salvati et al., 2019).

However, in recent years its practice has extended to other areas such as organization, schools and sports (De Allicon, 2020; Vickery and Dorjee, 2016; Doron et al., 2020; Emerson et al., 2020).

Viciana et al. (2018) in their review of scientific production in mindfulness for the decade 2007–2017 in the Web of Science, report a rather scarce production with respect to studies that focus on mindfulness within the university environment. They point out that, of the total of 652 articles in any language, only 96 would be related to the field of education. The research on mindfulness in the university environment is reduced to 24 articles, with a total sample of 4119 students.

The main results in the university population point to its effectiveness in promoting health, general well-being, increased life satisfaction and a decrease in states of anxiety and depression (Dvorakova et al., 2017). Benefits have also been noted in knowledge retention (Ramsburg and Youmans, 2014), and significant improvements in reading and science scores (Bakosh et al., 2016).

Mindfulness is defined as focusing attention in an intentional way on the present moment without judging (Kabat-Zinn, 1990). It has been conceptualized as having several elements such as acceptance, non-judging, and non-reactivity. This multi-faceted conceptualization of mindfulness allows for a more complete understanding of its relationships with other variables, and thus Baer et al. (2006) developed the Five Facet Mindfulness Questionnaire (*FFMQ*) that provides the most comprehensive coverage of the construct (Peters et al., 2015). The *FFMQ* arises from the exploratory factor analysis of the five most popular questionnaires that had been developed on mindfulness (Baer et al., 2006). It provides a broader assessment of mindfulness in its attentional, self-awareness and emotional regulation facets compared to other popular scales such as the *MAAS*, which evaluates only the component of mindfulness focused on the present by a single factor (Zhuang et al., 2017). In addition, it has demonstrated sensitivity to differences in mindfulness practice, allowing for discrimination between meditators and non-meditators (Cebolla et al., 2012). It consists of five dimensions: *observing* - the ability to perceive and recognize internal or external stimuli -, *describing* - to label with words the living experience -, *act with awareness*, *not-judging of inner experience* - equable vision before the thoughts, sensations or emotions that are perceived - and *not-reactivity to inner experience* - the latter refers to the distance with the experience and a period of time in which one does not react to the stimulus (Baer et al., 2006).

On an emotional level, mindfulness facilitates the development of a feeling of unconditional love, compassion and forgiveness toward ourselves and others (Gilbert, 2010; Neff, 2011; Siegel, 2007). With respect to oneself, self-compassion allows us to understand our own suffering from a broad and transcendent perspective, perceiving human imperfections and feeling empathy toward others (Wu et al., 2019). With respect to others, compassion would turn the suffering of others into a vehicle for connection, rather than isolation (Salzberg, 2011). McCullough et al. (1997) highlight the emotional dimension of empathy as the most important in the development of forgiveness toward others. This affective disposition creates a bond on which empathy is constructed, since it helps us to perceive our shared humanity. Worthington (2006) in his model REACH on forgiveness to others raises empathy as the core of the model, facilitating a positive emotional response and compassion toward a person or an event that hurt us. Thus, empathy seems to play a key role in forgiveness and compassion toward oneself and others, allowing the maintenance, reconciliation and repair of social relationships.

This paper aims to deepen the mechanisms through which the practice of mindfulness could foster our empathic ability. Empathy refers to the ability to be affected by and share the emotional state of another, to evaluate the reasons for the other’s state and to identify with the other, adopting their perspective (De Waal, 2008). It is an essential component of social cognition, contributing to and enhancing our ability to understand, respond to and adapt to the emotions of others, to be effective in emotional communication and to engage in prosocial behavior (Spreng et al., 2009). While there has been an evolution in definitions according to different approaches and perspectives, research emphasizes the distinction between cognitive and emotional components (Rankin et al., 2005). Nevertheless, it has been difficult to find consensus between the theoreticians and investigators about if the processes that are related to empathy – perspective taking, sympathy, personal anguish, emotional contagion or theory of mind – are part of an affective perception or a cognitive understanding (Spreng et al., 2009). Thus, it is evidenced a great difficulty in finding agreement on the interrelated processes that contribute to empathy. In this work we have conceptualized empathy as an emotional process mainly, not subordinated to a cognitive understanding, although this can help to improve understanding and behavior (Spreng et al., 2009).

Different studies on empathy seem to indicate that we are born with a biological predisposition to be empathic; nevertheless, its level of development is influenced and determined by the environment. To increase empathy in people would be a great benefit for society with respect to the diminution of antisocial behaviors (Winning and Boag, 2015). In this sense, to advance toward a society of solidarity and understanding it would be key to foster empathy.

Some research related to empathy suggests contradictory results regarding the influence of gender. The initial studies that examined these differences (Hoffman, 1977) show higher levels of empathy in women. Hoffman suggests that women have a greater tendency to put themselves in the place of the other, as well as a greater emotional resonance. Eisenberg and Lennon (1983) in an attempt to replicate these results found that women obtained higher scores when empathy was assessed through a questionnaire, but found no differences when physiological correlates or facial gestures were measured. This led to the assumption that the differences between men and women were due to gender role stereotypes, according to which when completing the questionnaires women tended to present themselves as more empathetic because this is what is expected of them regardless of the fact that there are no differences in reactivity. Later research supports these conclusions: Michalska et al. (2013) found that women scored higher than men on an empathy questionnaire, a difference that increased with age. However, they found no gender differences in pupillary reaction or neural or hemodynamic response, suggesting dissociation between the questionnaires and neurophysiological response. Recent studies have found gender differences in the influence of mindfulness on other constructs such as aggression or rumination (Peters et al., 2015; Eisenlohr-Moul et al., 2016) so the role of gender as a moderator in the effects of mindfulness on empathy will be explored. In recent years, mindfulness-based interventions have been proposed as a means to improve empathy responses (Fulton and Cashwell, 2015; Kemper and Khirallah, 2015; Foukal et al., 2016; Lamothe et al., 2016; Bellosta-Batalla et al., 2017). Empathy involves the ability to understand and share the feelings of others. Thus, the basic attentional processes involved in mindfulness (*observing, describing* and *acting with awareness*) could be key in the development of empathy. The capacity to be aware of what is happening in the present moment, and to *observe* and *describe* the emotions in oneself, would make the appearance of these capacities more likely in relationships with others. Jinpa et al. (2009) propose that people would be able to observe and describe their own suffering and would become aware of their desire to alleviate it, extending it to the suffering of others in a natural way. Also, the capacities of *non reactivity* and *non judging*, can be key in the empathic development since they would allow to distance themselves from the strong emotions when not reacting in excess, making possible to understand, to take care of and to respond properly to the other’s feelings (Wallmark et al., 2012). In this line, Bishop et al. (2004) raises mindfulness as a metacognitive capacity that orients people not only to their own affective state, but also to contextual stimuli in a non-reactive and acceptance way. Moreover, from a physiological point of view, it has been established that the practice of mindfulness causes changes in the same brain areas that are related to empathy: the prefrontal cortex, the anterior cingulate cortex and the anterior insula (Cahn and Polich, 2006; Lutz et al., 2008; Chiesa and Serretti, 2010; Shamay-Tsoory, 2011). Hölzel et al. (2011) found that the daily practice of 30 min of meditation increased the density of gray matter in the brain regions associated with empathy. Numerous studies have found this benefit (Greason and Cashwell, 2009; Birnie et al., 2010; De la Fuente-Anuncibay et al., 2019; Ngô, 2013; Shapiro et al., 1998). Nevertheless, several studies question this positive effect in the improvement of empathy when practicing mindfulness (Beddoe and Murphy, 2004; Dekeyser et al., 2008; Ridderinkhof et al., 2017).

Although there are many aspects to investigate (Fulton and Cashwell, 2015; Bibeau et al., 2016), there are theoretical as well as empirical evidences that support the connection between mindfulness and empathy (Jones et al., 2019). Most of studies explain this relation from correlations or regressions (Krasner et al., 2009; Fulton and Cashwell, 2015) being the therapeutic contexts, associated to health variables, and development of empathy skills in health professionals those who have centered the greater efforts (Aiken, 2006; Miró et al., 2015; Wang, 2006).

The popularization of mindfulness practice has increased in non-formal settings, but there is little research that attempts to explain the mechanisms through which mindfulness practice influences empathy outside of closed or structured programs (Barbosa et al., 2013; Jones et al., 2019), or the differential effects based on gender.

On the other hand, most of the research has been carried out in Anglo-Saxon areas. In this respect, De la Fuente-Anuncibay et al. (2019) in their study with English students, pointed out modifications in the mindfulness trait and their influence in the development of empathy, pointing out the necessity to extend it to samples of other countries, although we do not have evidences of this type in other contexts like the Spanish-speaking one.

The present study addresses the practice in an informal or non-therapeutic context (leisure, meditation centers, courses, etc.), which has been little studied previously, and focused on university students in Spain. The objectives set in this study focus on verifying that mindfulness practices produce changes in the cognitions of the mindfulness construct, which are those that influence the improvement of empathy.

**FIGURE 1:**
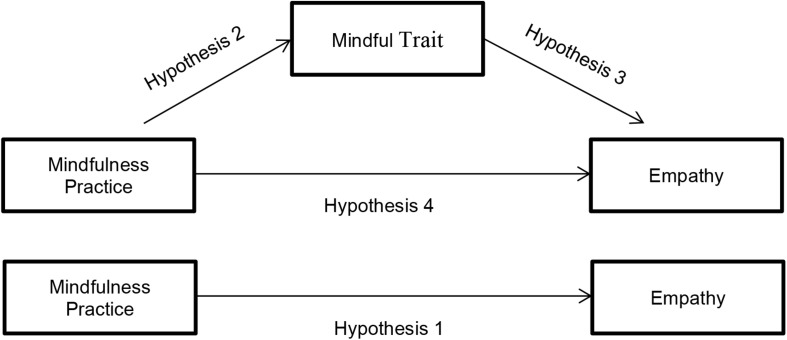
Hypothesized mediation model: Indirect effect of mindfulness practice on empathy through mindful trait and total effect of independent variable on dependent variable.

As illustrated in Figure 1, the total effect (*c*) refers to the relationship between mindfulness practice and empathy without controlling for the mediator (Hypothesis 1). The direct effect (*c*´) refers to the relationship between mindfulness practice and empathy after controlling for the mediator (Hypothesis 4). Finally, a total indirect effect (*ab*) refers to the role of the mediator in the relationship between mindfulness practice and empathy (Hypothesis 2 and 3).

The second objective will be to establish which are the factors of mindfulness trait that have more influence on the development of empathy in Spanish-speaking contexts. Mindfulness is a metacognitive factor that precedes other cognitive-affective processes, so we consider that it can function as a mediator and that the five facets of mindfulness could be influencing the increase of empathy skills. Thus, we expect to obtain similar results to the study by De la Fuente-Anuncibay et al. (2019) in English-speaking samples, in which *observing*, *describing*, and *not-reactivity* were predictors of empathy (Hypothesis 5).

As a third objective, because previous work has demonstrated gender differences in empathy (Michalska et al., 2013) and gender differences in the effects of mindfulness on other variables such as aggressiveness (Eisenlohr-Moul et al., 2016) we also explored gender as a moderator of the effects of mindfulness on the empathy (Hypothesis 6).

## Materials and Methods

### Participants

An convenience sample of 246 volunteer students from the University of Burgos (Spain) belonging to 7 degrees was selected: Degree in Social Education (*n* = 80), Degree in Pedagogy (*n* = 102), Degree in Early Childhood Education Teacher (*n* = 25), Master’s Degree in Inclusive Education and Society (*n* = 18), Course on Pedagogical and Didactic Qualification in Vocational Training (*n* = 15), and Master’s Degree in Teacher Training in Compulsory Secondary and Upper Secondary School Education (*n* = 6). The gender distribution was 35 men and 211 women with an average age of 22.23 years (*SD* = 4.76). The distribution of the sample presents a representative percentage of the university population in the area studied, characterized by a greater presence of women. In this sense, the OECD (2016) points out great differences according to the areas, greater in education (81%) with respect to health or social services (72%), these percentages being very similar to other European countries such as Ireland and the United Kingdom. The sampling was non-probabilistic convenience.

With regard to the practice of mindfulness, it was included in the group of people who practiced or had practiced mindfulness for non-therapeutic purposes in the last three years. The sample was divided into two groups according their responses to the question “Have you had any mindfulness training?”, separating those who had never practiced mindfulness and those who had practiced it in informal contexts, that is, outside closed programs with a predetermined number of sessions.

### Instruments

The scales used were the Five Facet Mindfulness Questionnaire (*FFMQ*) (Baer et al., 2006) and the Toronto Empathy Questionnaire (*TEQ*) (Spreng et al., 2009) selected for presenting adequate psychometric characteristics. The *FFMQ* was designed to measure mindfulness with psychometric guarantees from a factor analysis of the five most used scales that measured mindfulness trait, or the tendency to be more aware in daily life, consolidating a new 39-item questionnaire. It consists of five subscales: *observing*, *describing*, *act with awareness*, *not-judging of inner experience* and *not-reactivity to inner experience* (Baer et al., 2006). These are grouped into a general factor of second order that collects mindfulness cognitions in a single dimension. It has psychometric guarantees, adequate reliability, convergent and discriminatory validity, showing sensitivity to differences in the practice of mindfulness (Cebolla et al., 2012). Cronbach’s alpha was 0.888 for the *FFMQ* (*observing* = 0.803, *describing* = 0.926, *act with awareness* = 0.846, *not-judging of inner experience* = 0.861 and *not-reactivity to inner experience* = 0.640).

The *FFMQ* has been validated in many countries, including France, the Netherlands, Germany, China, Norway and Chile (Rodríguez, 2017).

The *TEQ* (Spreng et al., 2009) is a short questionnaire that measures empathy as a primary emotional process, as a broad construct. It is a reliable scale (Cronbach’s alpha = 0.69) robust, with internal consistency and test-retest reliability (Spreng et al., 2009). By means of a factorial analysis from the most used questionnaires to evaluate empathy, Spreng et al. (2009) obtained a one-dimensional factor that contemplates 16 items with contents on empathy such as emotional contagion and responses to the emotional state of the other or of help, altruism or activation of the sympathetic system.

Both scales have been used in previous studies with similar college populations (Quezada et al., 2012; Schmidt and Vinet, 2015; Mathad et al., 2017).

### Procedure

The data was collected by the research team on paper and participation was voluntary. The research was approved by the Bioethics Committee of the University of Burgos. The principles of anonymity and confidentiality of the data of the study participants were taken into account. To obtain information, students completed the questionnaires in a single phase of about 25–30 min.

## Results

### Preliminary Analysis

Means, standard deviations, skewness and kurtosis values, and correlations for major study variables are summarized in Table 1. We checked skewness and kurtosis in our mediators and criterion variables. Following Curran et al. (1996), skewness > ± 2 and kurtosis > ± 7 would indicate non-normal distribution. The results indicated that all measures were normally distributions, except *non-reactivity to inner experience* (Kurtosis = 8.18) dimension. However, as show Ng and Lin (2016) in your simulation, unlike classic methods, bootstrap methods do not rely on the assumption of asymptotic normality of the parameter estimates and offer better results. Even with a small sample size, bootstrap methods offer satisfactory control over Type I error and desirable statistical power (Ng and Lin, 2016). Moreover, PROCESS provides a method for testing of indirect effects that minimizes bias in results that can arise from non-normal sampling distributions (Hayes, 2018).

**TABLE 1:** Means, Standard Deviations, and Intercorrelations.

	M	SD	S	K	1	2	3	4	5	6
1. FFMQ	3.25	0.46	0.34	0.37						
2. Observing	3.21	0.71	0.16	–0.09	0.526***					
3. Describing	3.35	0.86	–0.06	–0.76	0.791***	0.337***				
4. Acting with Awareness	3.16	0.66	–0.14	–0.15	0.558***	–0.012	0.287***			
5. Non-judging of Inner Experience	3.39	0.71	–0.02	–0.21	0.630***	–0.022	0.351***	0.401***		
6. Non-reactivity to Inner Experience	3.11	0.66	1.27	8.18	0.651***	0.352***	0.420***	0.133*	0.270***	
7. TEQ	4.09	0.36	–0.25	–0.39	0.249***	0.270***	0.235***	0.118	0.051	0.087

S = Skewness, K = Kurtosis. *p < 0.05, ***p < 0.001.

As can be seen, empathy correlates positively with the mindfulness trait (*r* = 0.249, *p* < 0.0001). Of the dimensions of *FFMQ*, empathy was positively correlated with Observing (*r* = 0.270, *p* < 0.0001) and Describing (*r* = 0.235, *p* < 0.0001). The internal consistency indices (Cronbach’s alpha) of all variables in the study range from 0.640 to 0.926.

### Mediation Model

The first objective of this work is to confirm, from the total measurement of the mindfulness trait, the relationship between practicing mindfulness (in informal contexts) and improving empathy. Our hypothesis is that, in the presence of the mindfulness trait mediator, the direct effect of mindfulness practice on the improvement of empathy disappears.

We carried out a mediation analysis with the macro PROCESS for SPSS (Model 4), developed by Hayes (2018) in order to address the hypotheses of this study (Hypothesis 1 to H4). Mediation analyses test the effect of X (mindfulness practice) on Y (empathy) through the mediating variables (mindfulness trait). In this sense, mediation analysis allows us to assess how mindfulness practices influence empathy.

In our case, PROCESS generates coefficients using ordinary least squares (OLS) regression. We performed the analysis with 10000 bootstrap samples, with which it is not necessary to satisfy the assumptions of OLS (normality, lack of multicollinearity, etc.).

Furthermore, this technique allows introducing dichotomous variables into the model (in this case, having or not practicing mindfulness), without the restrictions and disadvantages that others, such as Structural Equation Modeling - SEM-, through Weighted Least Squares (WLS) estimation present.

Applying this method, we generate 95% bias-corrected confidence intervals for indirect effects. Hayes and Preacher (2010) recommend the use of bootstrapping techniques to obtain confidence limits for indirect effects. Bootstrapping is a non-parametric sampling technique that resamples several times, improving the power of a model, to estimate the indirect effect. The Bootstrapping IC is the most recommended way to evaluate indirect effects (Preacher et al., 2007). The indirect effect is statistically significant if the intervals do not include zero.

The results showed significant total effects of mindfulness practice on empathy (Hypothesis 1: β = 0.375, *p* = 0.043). The total indirect effects (ab) were statistically significant, since the 95% confidence interval (CI) of the point estimate did not cross zero (β = 0.214, BootSE = 0.085, Boot95% CI = 0.074, 0.404). So, there was a significant indirect pathway: the mindfulness practice was a significant predictor of mindfulness trait (Hypothesis 2: β = 0.916, *p* < 0.0001), which was itself a significant predictor of empathy (Hypothesis 3: β = 0.233, *p* = 0.0002). Considering the influence of the mediator, that is, considering this significant indirect route, the direct effect (c’) of mindfulness practice on empathy was not significant (Hypothesis 4: β = 0.161, *p* = 0.394), according to our approach (Table 2).

**TABLE 2:** Mediation model (PROCESS, Model 4).

	β	SE	p
Mediating variable model (DV: Mindful trait) Predictor			
Mindfulness practice (a)	0.916	3.189	0.0001
DV: Empathy Predictors			
Mindful trait (b)	0.233	0.020	0.0002
Mindfulness practice (c´)	0.161	1.093	0.394
Total effect			
Mindfulness practice (c)	0.375	1.067	0.043
Indirect effect (ab)	B	BootSE	Boot 95% CI
Mindfulness practice → Mindful trait → Empathy	0.214	0.085	[0.074, 0.404]

*Indirect effect of mindfulness practice of (X) on empathy (Y: TEQ) through changes in mindfulness trait (M: FFMQ). DV = dependent variable; CI = confidence interval.*

**FIGURE 2:**
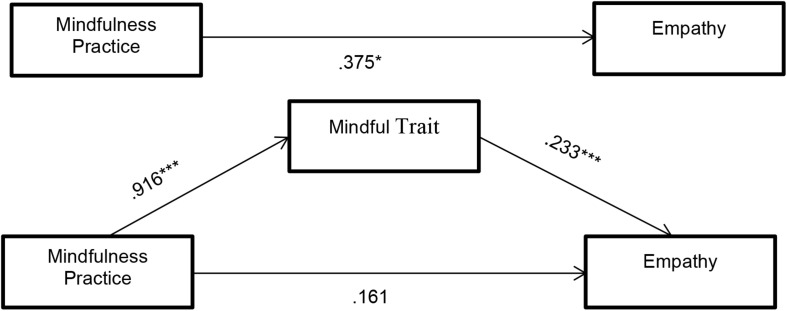
Results of the mediation model: Indirect effect of mindfulness practice on empathy through mindful trait and total effect of independent variable on dependent variable (standardized regression coefficients). *p < 0.05, ***p < 0.001.

As can be seen, all the hypotheses are confirmed as expected. Figure 2 shows the results of the mediation model, which indicate that the indirect effect is significant.

The second objective of the study is to analyze what mindfulness factors are mediating the overall effect between mindfulness practice and empathy.

**FIGURE 3:**
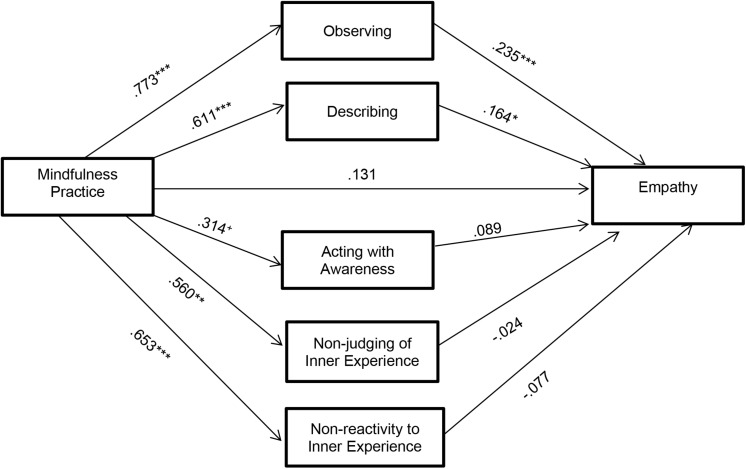
Results of the mediation model: Indirect effect of mindfulness practice on empathy through each mindfulness facets separately (Standardized regression coefficients). ***p < 0.001, *p < 0.05, ^+^*p* < 0.10.

The SPSS macro PROCESS, developed by Hayes (2018) was again used to check which *FFMQ* dimensions are mediating the relation between mindfulness practice and empathy (Figure 3). The indirect effect was also calculated using 10000 bootstrap samples for the bootstrap confidence intervals (CIs).

Table 3 shows the results of the indirect mediation routes for each of the mindfulness factors. These results indicated the indirect coefficient was significant (*B* = 0.244, *BootSE* = 0.09, *Boot95% CI* = 0.068, 0.448).

**TABLE 3:** Mediation model.

Indirect effect	B	BootSE	Boot 95% CI
MP → Observing → Empathy	0.179	0.073	[0.056, 0.338]
MP → Describing → Empathy	0.100	0.054	[0.008, 0.217]
MP → Acting with awareness → Empathy	0.028	0.034	[−0.019, 0.112]
MP → Non-judging of inner experience → Empathy	–0.013	0.044	[−0.108, 0.072]
MP → Non-reactivity to inner experience → Empathy	–0.050	0.052	[−0.169, 0.038]

*Indirect effect of mindfulness practice (X) on empathy (Y), presenting the mediating impact of each mindfulness facets (M). Partially standardized regression coefficients, MP = Mindfulness Practice.*

Only the indirect effects of the *observe* and *describe* dimensions are significant. The factors *acting with awareness*, *not-judging of inner experience* and *non-reactivity to inner experience*, would not work as mediating variables in the relationship between the practice of mindfulness and empathy.

Analyzing the significant results, the indirect effects indicate that the practice of mindfulness was a significant predictor of *observation* (*B* = 0.773, *p* < 0.001) and this was a significant predictor of *empathy* (*B* = 0.235, *p* < 0.001).

Also, it has been found that the practice of mindfulness was a significant predictor of the ability to *describe* (*B* = 0.611, *p* < 0.001) and this in turn was a significant predictor of *empathy* (*B* = 0.164, *p* < 0.05). The direct effect of the practice of mindfulness on the improvement of empathy also disappears (*B* = 0.131, *p* = 0.486), if the mediation of the *FFMQ* factors in that relation is taken into account.

### Moderated Mediation Model

**FIGURE 4:**
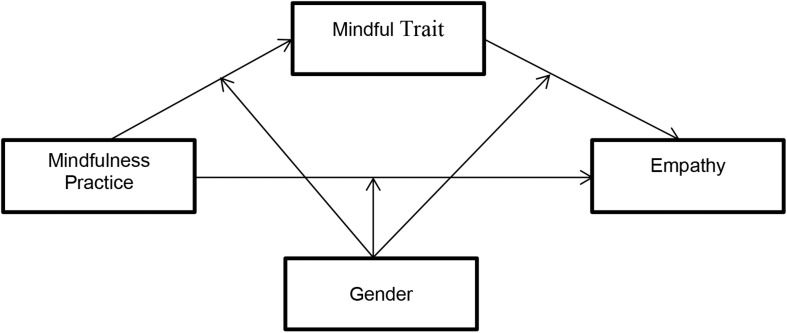
Moderated mediation model (model 59).

Finally, the present study expected that gender would moderate the indirect association between mindfulness practice and empathy via mindfulness trait. To test the moderated mediation hypothesis (Figure 4), the present study estimated parameters regression with PROCESS macro (Model 59) by Hayes (2018).

The results of the analysis allow us to conclude that gender was not a moderator in the proposed model. None of the interactions between the predictors variables and gender was statistically significant (Table 4).

**TABLE 4:** Results of the moderated mediation analysis (PROCESS, Model 59).

Predictor Variables	B	SE	p
	
	Mediating variable model (Mindfulness Trait)		
Mindfulness Practice	19.477	3.622	0.0000
Gender	15.590	9.176	0.091
Interaction Mindfulness Practice x Gender	–14.021	7.244	0.054
	
	**Dependent variable model (Empathy)**		
	
Mindfulness Trait	0.095	0.031	0.003
Mindfulness Practice (direct effect)	1.063	1.417	0.454
Gender	8.810	6.882	0.202
Interaction Mindfulness Trait x Gender	0.322	2.834	0.910
Interaction Mindfulness Practice x Gender	–0.049	0.063	0.434

*Unstandardized coefficients.*

## Discussion

The findings found in the present work seem to indicate that mindfulness practices developed in informal settings in Spanish-speaking contexts are a significant predictor of mindfulness trait, and that these are a significant predictor of the development of empathy. Therefore, our first research objective is fulfilled. Mindfulness would be a metacognitive capacity that influences later cognitive processes and, theoretically, also behavior, increasing in the individual the capacity to pay attention to their own affective state in the present moment, and also orienting them to contextual stimuli in a curious, open and accepting way (Bishop et al., 2004). According to these results, it seems that becoming aware and accepting one’s own emotions could have healthy effects on attending to the other person’s emotional experiences (Trautwein et al., 2014).

In relation to empathy, the meta-analysis made by Konrath et al. (2011) in which 72 studies on empathy of university students in the United States between 1979 and 2009 are analyzed, indicates that the present university students are 40% less empathic than those of twenty or thirty years ago, which is detrimental in attitudes of understanding, compassion, preoccupation or empathy toward other people. The importance of empathy in the educational field is undeniable.

There are numerous experimental researches that have observed an increase of empathy through the practice of mindfulness (Birnie et al., 2010; Shapiro et al., 2011; Gockel et al., 2012; Barbosa et al., 2013) pointing out improvements in university students after the application of the program.

In this regard, a growing body of studies on the benefits of mindfulness has begun to show its potential as an intervention strategy to improve mental health and overall performance. Research with university students points to the need to increase this body of study, confirming the need to investigate aspects of positive functioning (Viciana et al., 2018).

Empathy is an essential element in interpersonal relationships and a central competence in the university environment (Centeno and Fernandez, 2020). The ability to put oneself in the place of the other -intellectually and emotionally- is a key factor for a more peaceful and supportive society, where relationships with others are established in a healthy way (Moya-Albiol, 2014). Research on the different forms of educating in empathy is seen as an area of special relevance due to its social and individual implications. In this sense, mindfulness is presented as a promising alternative since the attitudes and personal dispositions that it develops are intimately related to empathy. Empathy facilitates an approach to subjectivity and the way of understanding and feeling the world of others, regardless of whether it is different from our own. This approach is based on an attitude of acceptance and absence of judgment, which guarantees a sincere vision in which the image of the other is not altered by our own subjectivity. In the emotional sphere, empathy favors the establishment of an affective disposition of help. Furthermore, mindfulness helps us to recognize more subtle emotional states in ourselves and in others, facilitating our understanding of human emotions. Finally, the ability not to get carried away allows a distance to be established between others affectivity and one’s own, thus avoiding a fusion between the two. Further research is needed to establish the mechanisms through which mindfulness practices in university students improve empathy or other personal and social competencies that contribute to the development of their strengths and potential.

Despite the increase in the practice of mindfulness in informal or leisure contexts, there are hardly any studies on the effects for those who practice it for this purpose, since research has focused on therapeutic or formal contexts. The results of our study confirm that even informal practice of mindfulness can be considered a quality experience, because of the benefits it brings to the people who practice it, such as modifications in their mindfulness components or the increase of empathy. Gim (2009) consider contemplative practice as the essence of leisure because it creates a special experience that provides choice, freedom, tranquility, flow, and satisfaction. In line with our study, positive results are also obtained not only through greater internal balance but also by increasing well-being and a positive sense of health. With regard to the second objective, the dimensions of *FFMQ* that function as mediators between the practice of mindfulness and the improvement of empathy we have found two: *observing* and *describing*. No mediation on empathy was found in the dimensions of *acting with awareness*, *non-judging of inner experience*, and *non-reactivity to inner experience*. The current conception is that mindfulness consists of two distinct cognitive processes: attention focused on the present and acceptance of emotions. These two processes are often measured with *FFMQ* (Baer et al., 2006; Jones et al., 2019). The three *FFMQ* factors that shape present-centered attention are *observing* (attention to internal and external stimuli), *describing* (labeling and expressing experiences), and *acting with awareness* (attention in the present to one’s own behaviors rather than responding automatically). The two *FFMQ* factors that make up the acceptance of emotions are *non-judging* (adopting an unevaluated stance toward thoughts and emotions), and *non-reactivity* (letting emotions flow without being trapped by them). According to our results, the improvement of empathy due to informal mindfulness practices is due to processes related mainly to the attention focused on the present. And for this, it would not be necessary to *act with awareness*, but it would be enough to put the focus on the stimulation we experience and be able to label it and express it correctly. It seems clear then, that paying attention to internal processes has an influence on the sensitivity to other people’s needs or concerns.

If *observing* and *describing* one’s own emotions increases attention to the experiences of others, that is to say, empathy, other research (Neff and Beretvas, 2013) found that people who reported that they could label their own feelings correctly also reported that they could help others overcome difficult emotions, as well as the relationship between the degree to which people are kind to themselves and the kindness they have to another person.

In the same line, we find results in the works of Jones et al. (2019) and De la Fuente-Anuncibay et al. (2019) in English-speaking samples. In both studies they use *FFMQ* as a measure of mindfulness, with practice being the predictor variable. The scale used by Jones et al. (2019) to evaluate empathy was the Interpersonal Reactivity Scale (Davis, 1994) in a sample of American university students with little or no experience in the practice of mindfulness. Their results agree in that *observing* and *describing* were related to the improvement in the empathic abilities. On the other hand, they pointed out that adopting a non-evaluative posture toward emotions (*Non-judging*) negatively predicted empathy. On the other hand, the scale used by De la Fuente-Anuncibay et al. (2019) in a sample of English university students who made informal practices of mindfulness, was the *TEQ* (Spreng et al., 2009). Their results informed that, in addition to the dimensions of *observing* and *describing*, it influenced the dimension of not allowing to be caught by the own emotions (*Non-reactivity*) in the increase of empathy. We can suppose that the differences in some of our results, with respect to the work of Jones et al. (2019) are due to the scales used to evaluate empathy. The *TEQ* evaluates empathy as an emotional process and the content of the items has to do with the identification and understanding of others’ emotions, physiological activation, altruism, and the frequency of empathic and prosocial behaviors. IRI evaluates empathy around four factors: Perspective taking, Fantasy, Empathic concern and Personal distress. On the other hand, the only difference of the present work with the mentioned one of De la Fuente-Anuncibay et al. (2019) is that that one was carried out in an Anglo-Saxon country and this one in a Spanish-speaking context.

We have not found other investigations that use mindfulness trait as mediators that influence the increase of empathy, being this study a pioneer in this context.

Regarding the third objective, gender does not seem to function as a moderator between the practice of mindfulness and the changes it predicts in the mindfulness trait. Neither does it moderate between mindfulness trait and empathy, nor between mindfulness practice and empathy. This is consistent with previous research (Dean et al., 2017) that used structured mindfulness programs and did not find a different gendered functioning in the development of empathy. Our results support the idea that mindfulness training is equally effective in men and women, and has the same effects on empathy in both genders.

The findings of this paper should be interpreted with caution because of its limitations. Firstly, this study is transversal and therefore does not allow for the establishment of causality. Accordingly, researches with longitudinal designs are needed to provide more evidence for the hypotheses being raised. Another limitation could be reinforced by the use of random sampling, which is more powerful than the non-probability sampling used, thus avoiding the risk of bias. Finally, the limitation of the use of self-reports for the assessment of mindfulness and empathy should be established. Although both instruments are designed on the basis of factor analyses of the most commonly used questionnaires in both constructs and have sufficient psychometric guarantees, some authors point out deficiencies in them such as the influence of subjective perception or problems in establishing real change. Thus, situational assessments or the use of biological markers for empathy (Bellosta-Batalla et al., 2017; Breithaupt, 2009) or objective monitoring (Quintana and Rivera, 2012) and the use of behavioral measures of mindfulness practice (Grossman, 2011) are proposed.

## Data Availability Statement

The raw data supporting the conclusions of this article will be made available by the authors, without undue reservation.

## Ethics Statement

The studies involving human participants were reviewed and approved by Bioethics Committee of the University of Burgos (IR 15/2018). The patients/participants provided their written informed consent to participate in this study.

## Author Contributions

RF-A, ÁG-B, DO-S, and JP-R performed the conceptualization, methodology, formal analysis, writing original draft preparation, review and editing, visualization, supervision, project administration, investigation, resources, and data curation.

## Conflict of Interest

The authors declare that the research was conducted in the absence of any commercial or financial relationships that could be construed as a potential conflict of interest.

## References

[B1] Aiken G. A. (2006). *The Potential Effect of Mindfulness Meditation On the Cultivation of Empathy in Psychotherapy: A Qualitative Inquiry.* Dissertation, University of Saybrook, California.

[B2] Baer R. A. Smith G. T. Hopkins J. Krietemeyer J. Toney L. (2006). Using self-report assessment methods to explore facets of mindfulness. *Assessment* 13 27–45. 10.1177/1073191105283504 16443717

[B3] Bakosh L. S. Snow R. M. Tobias J. M. Houlihan J. L. Barbosa-Leiker C. (2016). Maximizing mindful learning: mindful awareness intervention improves elementary school students’ quarterly grades. *Mindfulness* 7 59–67. 10.1007/s12671-015-0387-6

[B4] Barbosa P. Raymond G. Zlotnick C. Wilk J. Toomey R. Mitchell J. (2013). Mindfulness-based stress reduction training is associated with greater empathy and reduced anxiety for graduate healthcare students. *Educ. Health* 26 9–14. 10.4103/1357-6283.112794 23823667

[B5] Barcaccia B. Baiocco R. Pozza A. Pallini S. Mancini F. Salvati M. (2019). The more you judge the worse you feel. A judgemental attitude towards one’s inner experience predicts depression and anxiety. *J. Individ. Diff.* 138 33–39. 10.1016/j.paid.2018.09.012

[B6] Beddoe A. E. Murphy S. O. (2004). Does mindfulness decrease stress and foster empathy among nursing students? *J. Nurs. Educ. Pract.* 43 305–312. 10.3928/01484834-20040701-07 15303583

[B7] Bellosta-Batalla M. Pérez-Blasco J. Cebolla A. Moya-Albiol L. (2017). Empatía y mindfulness. *Convergencia teórica*. *Rev. Latinoamericana Psicol. Posit.* 3 34–44.

[B8] Bibeau M. Dionne F. Leblanc J. (2016). Can compassion meditation contribute to the development of psychotherapists’ empathy? A review. *Mindfulness* 7 255–263. 10.1007/s12671-015-0439-y

[B9] Birnie K. Speca M. Carlson L. E. (2010). Exploring self-compassion and empathy in the context of mindfulness-based stress reduction (MBSR). *Stress Health* 26 359–371. 10.1002/smi.1305

[B10] Bishop S. R. Lau M. Shapiro S. Carlson L. Anderson N. D. Carmody J. (2004). Mindfulness: a proposed operational definition. *Clin. Psychol. Sci.* 11 230–241. 10.1093/clipsy.bph077

[B11] Breithaupt F. (2009). *Kulturen Der Empathie.* Frankfurt am Main: Suhrkamp.

[B12] Cahn B. R. Polich J. (2006). Meditation states and traits: EEG, ERP, and neuroimaging studies. *Psychol. Bull.* 132 180–211. 10.1037/0033-2909.132.2.180 16536641

[B13] Cebolla A. Garcia-Palacios A. Soler J. Guillén V. Baños R. Botella C. (2012). Psychometric properties of the Spanish validation of the five facets of mindfulness questionnaire (FFMQ). *Eur. J. Psychiatry* 26 118–126. 10.4321/s0213-61632012000200005 31832788

[B14] Centeno R. P. R. Fernandez K. T. (2020). Effect of mindfulness on empathy and self-compassion: an adapted MBCT program on filipino college students. *Behav. Sci.* 10:61. 10.3390/bs10030061 32120924 PMC7139462

[B15] Chiesa A. Serretti A. (2010). A systematic review of neurobiological and clinical features of mindfulness meditations. *Psychol. Med.* 40 1239–1252. 10.1017/s0033291709991747 19941676

[B16] Coholic D. A. Eys M. (2015). Benefits of an arts-based mindfulness group intervention for vulnerable children. *Child Adolesc. Soc. Work J.* 33 1–13. 10.1007/s10560-015-0431-3

[B17] Curran P. J. West S. G. Finch J. F. (1996). The robustness of test statistics to nonnormality and specification error in confirmatory factor analysis. *Psych. Meth.* 1 16–29. 10.1037/1082-989x.1.1.16

[B18] Davis M. H. (1994). *Empathy: A Social Psychological Perspective.* Boulder, CO: Westview.

[B19] De Allicon K. (2020). A mindfulness toolkit to optimise incident management and business continuity exercises. *J. Bus. Contin. Emer. Plan.* 13 220–229. 32093813

[B20] De la Fuente-Anuncibay R. González-Barbadillo A. Cubo E. González-Bernal J. Pizarro Ruiz J. P. (2019). Mediating effect of mindfulness cognition on the development of empathy in a university context. *PLoS One* 14:e0215569. 10.1371/journal.pone.0215569 30998744 PMC6472790

[B21] De Waal F. B. (2008). Putting the altruism back into altruism: the evolution of empathy. *Annu. Rev. Psychol.* 59 279–300. 10.1146/annurev.psych.59.103006.093625 17550343

[B22] Dean S. Foureur M. Zaslawski C. Newton-John T. Yu N. Pappas E. (2017). The effects of a structured mindfulness program on the development of empathy in healthcare students. *Nursingplus Open* 3 1–5. 10.1016/j.npls.2017.02.001

[B23] Dekeyser M. Raes F. Leijssen M. Leysen S. Dewulf D. (2008). Mindfulness skills and interpersonal behaviour. *J. Individ. Differ.* 44 1235–1245. 10.1016/j.paid.2007.11.018

[B24] Doron J. Rouault Q. Jubeau M. Bernier M. (2020). Integrated mindfulness-based intervention: effects on mindfulness skills, cognitive interference and performance satisfaction of young elite badminton players. *J. Sport Exerc. Psychol.* 47:101638 10.1016/j.psychsport.2019.101638

[B25] Dvorakova K. Kishida M. Li J. Elavsky S. Broderick P. C. Agrusti M. R. (2017). Promoting healthy transition to college through mindfulness training with first-year college students: pilot randomized controlled trial. *J. Am. Coll. Health* 65 259–267. 10.1080/07448481.2017.1278605 28076182 PMC5810370

[B26] Eisenberg N. Lennon R. (1983). Sex differences in empathy and related capacities. *Psychol. Bull.* 94 100–131. 10.1037/0033-2909.94.1.100

[B27] Eisenlohr-Moul T. A. Peters J. R. Pond R. S. DeWall C. N. (2016). Both trait and state mindfulness predict lower aggressiveness via anger rumination: a multilevel mediation analysis. *Mindfulness* 7 713–726. 10.1007/s12671-016-0508-x 27429667 PMC4943669

[B28] Emerson L. M. de Diaz N. N. Sherwood A. Waters A. Farrell L. (2020). Mindfulness interventions in schools: integrity and feasibility of implementation. *Int. J. Behav. Dev.* 44 62–75. 10.1177/0165025419866906

[B29] Foukal M. D. Lawrence E. C. Jennings P. A. (2016). Mindfulness and mentoring satisfaction of college women mentoring youth: implications for training. *Mindfulness* 7 1327–1338. 10.1007/s12671-016-0574-0

[B30] Fulton C. L. Cashwell C. S. (2015). Mindfulness-based awareness and compassion: predictors of counselor empathy and anxiety. *Couns. Educ. Sup.* 54 122–133. 10.1002/ceas.12009

[B31] Gilbert P. (2010). *Compassion-Focused Therapy: Distinctive Features.* London: Routledge.

[B32] Gim J. (2009). Toward a quality leisure experience: the practice of mindfulness. *World Leis J.* 51 105–109. 10.1080/04419057.2009.9674592

[B33] Gockel A. Burton D. James S. Bryer E. (2012). Introducing mindfulness as a self-care andclinical training strategy for beginning social work students. *Mindfulness* 4 1–11. 10.1007/s12671-012-0134-1

[B34] Greason P. B. Cashwell C. S. (2009). Mindfulness and counseling self-efficacy: the mediating role of attention and empathy. *Couns. Educ. Sup.* 49 2–19. 10.1002/j.1556-6978.2009.tb00083.x

[B35] Grossman P. (2011). Defining mindfulness by how poorly I think I pay attention during everyday awareness and other intractable problems for psychology’s (re)invention of mindfulness: comment on Brown et al. (2011). *Psychol. Assess.* 23 1034–1040. 10.1037/a0022713 22122674

[B36] Hayes A. F. (2018). *Introduction to Mediation, Moderation and Conditional Process Analysis*, 2nd Edn New York, NY: The Guilford Press.

[B37] Hayes A. F. Preacher K. J. (2010). Quantifying and testing indirect effects in simple mediation models when the constituent paths are nonlinear. *Multiv. Behav. Res.* 45 627–660. 10.1080/00273171.2010.498290 26735713

[B38] Hoffman M. L. (1977). Sex differences in empathy and related behaviors. *Psychol. Bull.* 84 712–722. 10.1037/0033-2909.84.4.712 897032

[B39] Hölzel B. K. Carmody J. Vangel M. Congleton C. Yerramsetti S. M. Gard T. (2011). Mindfulness practice leads to increases in regional brain gray matter density. *Psychiatry Res. Neuroimaging* 191 36–43. 10.1016/j.pscychresns.2010.08.006 21071182 PMC3004979

[B40] Jinpa T. Rosenberg E. McGonigal K. Cullen M. Goldin P. Ramel W. (2009). *Compassion Cultivation Training (CCT): An Eight-Week Course on Cultivating Compassionate Heart and Mind.* Stanford, CA: Center for Compassion and Altruism Research and Education, Stanford University.

[B41] Jones S. M. Bodie G. D. Hughes S. D. (2019). The impact of mindfulness on empathy, active listening, and perceived provisions of emotional support. *Commun. Res.* 46 838–865. 10.1177/0093650215626983

[B42] Kabat-Zinn J. (1990). *Full Catastrophe Living: Using The Wisdom of Your Body and Mind to Face Stress, Pain and Illness.* New York, NY: Delacorte.

[B43] Kemper K. J. Khirallah M. (2015). Acute effects of online mind–body skills training on resilience, mindfulness, and empathy. *J. Evid. Complement. Altern. Med.* 20 247–253. 10.1177/2156587215575816 25783980

[B44] Konrath S. H. O’Brien E. H. Hsing C. (2011). Changes in dispositional empathy in American college students over time: a meta-analysis. *Pers. Soc. Psychol. Rev.* 15 180–198. 10.1177/1088868310377395 20688954

[B45] Krasner M. S. Epstein R. M. Beckman H. Suchman A. L. Chapman B. Mooney C. J. (2009). Association of an educational program in mindful communication with burnout, empathy, and attitudes among primary care physicians. *JAMA* 302 1284–1293. 10.1001/jama.2009.1384 19773563

[B46] Lamothe M. Rondeau É Malboeuf-Hurtubise C. Duval M. Sultan S. (2016). Outcomes of MBSR or MBSR-based interventions in health care providers: a systematic review with a focus on empathy and emotional competencies. *Complement Ther. Med.* 24 19–28. 10.1016/j.ctim.2015.11.001 26860797

[B47] Lutz A. Brefczynski-Lewis J. Johnstone T. Davidson R. J. (2008). Regulation of the neural circuitry of emotion by compassion meditation: effects of meditative expertise. *PLoS One* 3:e1897. 10.1371/journal.pone.0001897 18365029 PMC2267490

[B48] Mathad M. D. Pradhan B. Rajesh S. K. (2017). Correlates and predictors of resilience among baccalaureate nursing students. *J. Clin. Diagn. Res. JCDR* 11:JC05. 10.7860/JCDR/2017/24442.9352 28384889 PMC5376833

[B49] McCullough M. E. Worthington E. L. Jr. Rachal K. C. (1997). Interpersonal forgiving in close relationships. *J. Pers. Soc. Psychol.* 73 321–336. 10.1037/0022-3514.73.2.321 9248052

[B50] Michalska K. J. Kinzler K. D. Decety J. (2013). Age-related sex differences in explicit measures of empathy do not predict brain responses across childhood and adolescence. *Dev. Cogn. Neurosci.* 3 22–32. 10.1016/j.dcn.2012.08.001 23245217 PMC6987715

[B51] Miró M. T. Ibáñez I. Felipe I. García N. M. (2015). Entrenamiento en “open mindfuness: un estudio piloto. *R. Psicot.* 26 145–159. 10.33898/rdp.v26i102.51

[B52] Moya-Albiol L. (2014). *La Empatia: Entenderla Para Entender a Los Demás.* Barcelona: Plataforma Actual.

[B53] Neff K. D. (2011). *Self-Compassion.* New York, NY: William Morrow.

[B54] Neff K. D. Beretvas S. N. (2013). The role of self-compassion in romantic relationships. *Self Identity* 12 78–98. 10.1080/15298868.2011.639548

[B55] Ng M. Lin J. (2016). Testing for mediation effects under non-normality and heteroscedasticity: a comparison of classic and modern methods. *Int. J. Q. Res. Educ.* 3 24–40.

[B56] Ngô T. L. (2013). Review of the effects of mindfulness meditation on mental and physical health and its mechanisms of action. *Santé ment Que* 38 19–34. 10.7202/1023988ar 24719001

[B57] OECD (2016). *). Panorama de La Educación. Indicadores de la OCDE 2016.* Madrid: Ministerio de Educación, Cultura y Deporte.

[B58] Peters J. R. Smart L. M. Eisenlohr-Moul T. A. Geiger P. J. Smith G. T. Baer R. A. (2015). Anger rumination as a mediator of the relationship between mindfulness and aggression: the utility of a multidimensional mindfulness model. *J. Clin. Psychol.* 71 871–884. 10.1002/jclp.22189 25919798 PMC12063192

[B59] Preacher K. J. Rucker D. D. Hayes A. F. (2007). Addressing moderated mediation hypotheses: theory, methods, and prescriptions. *Multiv. Behav. Res.* 42 185–227. 10.1080/00273170701341316 26821081

[B60] Quezada C. Pablo Robledo J. Roman D. Cornejo C. (2012). Empathy and pitch convergence. *RLA* 50 145–165.

[B61] Quintana M. Rivera O. (2012). Mindfulness training online for stress reduction, a global measure. *Stud. Health Techonol. Informatics* 181 143–148. 10.3233/978-1-61499-121-2-143 22954845

[B62] Ramsburg J. T. Youmans R. J. (2014). Meditation in the higher-education classroom: meditation training improves student knowledge retention during lectures. *Mindfulness* 5 14–31. 10.1007/s12671-013-0199-5

[B63] Rankin K. P. Kramer J. H. Miller B. L. (2005). Patterns of cognitive and emotional empathy in frontotemporal lobar degeneration. *Cogn. Behav. Neurol* 18 28–36. 10.1097/01.wnn.0000152225.05377.ab 15761274

[B64] Ridderinkhof A. de Bruin E. I. Brummelman E. Bögels S. M. (2017). Does mindfulness meditation increase empathy? An experiment. *Self Identity* 16 251–269. 10.1080/15298868.2016.1269667

[B65] Rodríguez N. (2017). Mindfulness: instrumentos de evaluación. Una revisión bibliográfica. *PSOCIAL* 3 46–65.

[B66] Salvati M. Chiorri C. Baiocco R. (2019). The relationships of dispositional mindfulness with sexual prejudice and internalized sexual stigma among heterosexual and gay/bisexual men. *Mindfulness* 10 2375–2384. 10.1007/s12671-019-01215-6

[B67] Salzberg S. (2011). *Real Happiness. The Power of Meditation.* New York, NY: Workman Publishing.

[B68] Schmidt C. Vinet E. V. (2015). Mindfulness: validation of the five facet mindfulness questionnaire (FFMQ) in chilean university students. *Terapia Psicol.* 33 93–101.

[B69] Shamay-Tsoory S. G. (2011). The neural bases for empathy. *Neuroscientist* 17 18–24. 10.1177/1073858410379268 21071616

[B70] Shapiro S. L. Brown K. W. Thoresen C. Plante T. G. (2011). The moderation of mindfulness-based stress reduction effects by trait mindfulness: results from a randomized controlled trial. *J. Clin. Psychol.* 67 267–277. 10.1002/jclp.20761 21254055

[B71] Shapiro S. L. Schwartz G. E. Bonner G. (1998). Effects of mindfulness-based stress reduction on medical and premedical students. *J. Behav. Med.* 21 581–599. 10.1023/A:1018700829825 9891256

[B72] Siegel D. J. (2007). *The Mindful Brain. Reflection and Attunement in the Cultivation of Well-Being.* New York, NY: W.W. Norton & Company. 10.1176/appi.ajp.2007.07081292 22688157

[B73] Spreng R. N. McKinnon M. C. Mar R. A. Levine B. (2009). The Toronto Empathy Questionnaire: scale development and initial validation of a factor-analytic solution to multiple empathy measures. *J. Pers. Assess.* 91 62–71. 10.1080/00223890802484381 19085285 PMC2775495

[B74] Ted Ng K. S. Chan H. Y. Wee S. T. Goh L. G. Nur F. Ying Tan C. T. (2016). Mindful awareness practice (MAP) to improve the cognition of singaporean elderly with mild cognitive impairment (MCI): a randomized controlled trial (rct). *Alz Dem.* 12 1180–1181. 10.1016/j.jalz.2016.07.118

[B75] Trautwein F.-M. Naranjo J. R. Schmidt S. (2014). “Meditation effects in the social domain: self-other connectedness as a general mechanism?,” in *Meditation–Neuroscientific Approaches and Philosophical Implications*, eds Schmid S. Walach H. (Cham: Springer), 175–198. 10.1007/978-3-319-01634-4_10

[B76] Viciana V. Fernández A. Linares M. Espejo T. Puertas P. Chacón R. (2018). Los Estudios Universitarios y el Mindfulness. Una revisión Sistemática. *RINACE* 16 119–135. 10.15366/reice2018.16.1.008

[B77] Vickery C. E. Dorjee D. (2016). Mindfulness training in primary schools decreases negative affect and increases meta-cognition in children. *Front. Psychol.* 6:2025. 10.3389/fpsyg.2015.02025 26793145 PMC4709470

[B78] Wallmark E. Safarzadeh K. Daukantaitë D. Maddux R. E. (2012). Promoting altruism through meditation: an 8-week randomized controlled pilot study. *Mindfulness* 4 223–234. 10.1007/s12671-012-0115-4

[B79] Wang S. J. (2006). *Mindfulness Meditation: Its Personal and Professional Impact on Psychotherapists.* Dissertation, University of Capella, Minneapolis.

[B80] Winning A. P. Boag S. (2015). Does brief mindfulness training increase empathy? The role of personality. *J. Individ. Differ.* 86 492–498. 10.1016/j.paid.2015.07.011

[B81] Worthington E. (2006). *Perdón y Reconciliación.* Nueva York: Routledge.

[B82] Wu Q. Chi P. Zeng X. Lin X. Du H. (2019). Roles of anger and rumination in the relationship between self-compassion and forgiveness. *Mindfulness* 10 272–278. 10.1007/s12671-018-0971-7

[B83] Zeng X. Chan V. Y. L. Liu X. Oei T. P. S. Leung F. Y. K. (2017). The four immeasurables meditations: differential effects of appreciative joy and compassion meditations on emotions. *Mindfulness* 8 949–959. 10.1007/s12671-016-0671-0

[B84] Zhuang K. Bi M. Li Y. Xia Y. Guo X. Chen Q. (2017). A distinction between two instruments measuring dispositional mindfulness and the correlations between those measurements and the neuroanatomical structure. *Sci. Rep.* 7 1–9. 28740242 10.1038/s41598-017-06599-w PMC5524689

